# Work ability and health of security guards at a public University: a cross-sectional study**^1^**

**DOI:** 10.1590/1518-8345.0616.2725

**Published:** 2016-07-25

**Authors:** Marluce Rodrigues Godinho, Aldo Pacheco Ferreira, Rosangela Maria Greco, Liliane Reis Teixeira, Maria Teresa Bustamante Teixeira

**Affiliations:** 2 Doctoral Student, Escola Nacional de Saúde Pública Sergio Arouca, Fundação Oswaldo Cruz, Rio de Janeiro, RJ, Brazil.; 3 PhD, Researcher, Centro de Estudos da Saúde do Trabalhador e Ecologia Humana, Fundação Oswaldo Cruz, Rio de Janeiro, RJ, Brazil.; 4 PhD, Professor, Faculdade de Enfermagem, Universidade Federal de Juiz de Fora, Juiz de Fora, MG, Brazil.; 5 PhD, Researcher, Centro de Estudos da Saúde do Trabalhador e Ecologia Humana, Fundação Oswaldo Cruz, Rio de Janeiro, RJ, Brazil.; 6 PhD, Professor, Faculdade de Medicina, Universidade Federal de Juiz de Fora, Juiz de Fora, MG, Brazil.

**Keywords:** Occupational Health, Work Ability Evaluation, Work

## Abstract

**Objective:**

to evaluate the work ability and health status of security guards at a public University.

**Methods:**

a cross-sectional, descriptive, and analytical study was carried with 119 security guards. The following instruments were used: Work Ability Index (WAI), Patient Health Questionnaire (PHQ-9), International Physical Activity Questionnaire (IPAQ, short), Alcohol Use Disorders Identification Test (AUDIT), Medical Outcomes Study (MOS), and Demand-Control-Support (DCS). Descriptive statistics were used to describe the study samples and the Spearman's coefficient correlation was performed to assess the WAI. Significance level was set at 5%.

**Results:**

samples were composed by men; the mean age was 54.9 years (SD=5.7); 80% had partners, and 75% had basic education. The majority (95%) had only one job, the average length of service was 24.8 years (SD=11), ranging from 3 to 43 years. 88.9% worked ≤40 hours and 75% did not work at night shift or rotating shifts. The average score given to work ability was good (40.7 points), with significant correlation to social support at work (p-value=0.002), health conditions (p-value=0.094), and depression symptoms (p-value=0.054).

**Conclusion:**

this study showed that many characteristics might affect the work ability scores. Considering the results, we note that healthy life habits and a reorganization of work environments should be encouraged.

## Introduction

The concept of work ability expresses: the evaluation of the productive capabilities of a worker, the worker's health, and his psychological resources^1^. It has been defined as the degree in which a worker, given his health status, is physically and mentally able to cope with the demands at work. In this approach, the primary focus of the work ability is on the worker's health^1^. Therefore, the physical and mental health status plays a role in the work ability, but may not be a determinant of it. Different factors, including the physical and psychosocial demands at work, the worker's mental and physical capabilities, and the lifestyle factors may influence the work ability. The imbalance between these determinants and the worker's health may lead to productivity loss at work, sickness, and work-related disability^2^.

Studies about the work ability has gained importance in the last years due to the aging of the active population worldwide. Challenges of the aging process may be related with the high speed in which the active population is aging worldwide, and with the absence of improvement in the living conditions after retirement^3^. In this context, determining the work ability has increasingly gained relevance and has recently been used as an important tool to predict workers' capabilities to perform their tasks in the future^2^. In addition, previous studies showed that maintaining a good work ability is associated with a prolonged working life, and with reduced losses on the workforce^2^ ^,^ ^4^.

In Brazil, studies on work ability began to emerge in the late nineties and since then, several authors have been dedicated to study this issue^5^. However, according to a systematic review^5^, these studies are case-by-case and address specific groups of workers (e.g., health professionals, production line workers, electricians, firefighters, administrative workers and forensic servers). Therefore, this review showed that there are still many professional categories to be assessed with regard to the work ability.

In this study, we aimed to address the security guards, which represents a professional category that is still understudied in the occupational health field. Security guards are professionals responsible for guaranteeing the security and the physical integrity of employees, workers and visitors, in public institutions, like universities. These features of the security guard allow this professional category to make use of firearms, if necessary. Furthermore, because of these specificities, the security guards are required to be constantly alert and react rapidly at any circumstance that threatens or violates the security. A preserved physical and psychological health are therefore, requirements for the vigilant profession. In this context, the study of work ability on security guards may contribute to improve their working conditions, as well as their health, which ultimately may influence on their quality of life.

Scientific evidences shows that the work ability may be influenced by several factors, including the health state, social and demographic characteristics, the lifestyle, and factors related to work. The wear and tear deriving from demands of work can be linked to chronic and acute physiological responses, psychological reactions and behavioral changes, with the possibility of decreased work ability and triggering work-related diseases. Otherwise, requirements that are characterized as positive could promote and protect the health and the work ability, irrespective of the age of the worker^1^. Authors^6^ point out that the promotion of functional capacity of workers may contribute for the quality of life after retirement and to reduce costs in maintaining the health in elderly.

Regarding health conditions and lifestyle habits, previous studies showed that health conditions are critical with regard to quality of life and work ability of individuals^7^ ^-^ ^8^. This implies that the evaluation of the work ability should include not only medical factors but also non-medical factors responsible for a decreased ability to perform the job^7^. Therefore, this study aimed to evaluate the work ability and health status of security guards who work in a public University. Evidence provided by our study may enable us to recommend actions to reduce, control and prevent the decline in work ability and actions for its improve.

## Methods

A cross-sectional study was conducted using data collected at the Federal University of Juiz de Fora, Minas Gerais, Brazil. The current study sample consisted of 119 security guards who perform their usual functions at University. The participants were sampled for the study between February 2012 and August 2013. For sample selection, the following inclusion criteria were applied: to have a technical-administrative permanent position in the education area; to be in active exercise of the function for a minimum of 10 years. The exclusion criteria were: to be on medical leave, to be licensed to training/qualification professional or to be an outsourced worker.

Data were collected by means of self-report questionnaires that were applied at the participant's workplace. The questionnaire used for data collection was 22 pages in total and the application time of each survey ranged from 20 to 30 minutes. Questionnaires that were returned with incomplete filling or in blank were considered losses. The dependent variable work ability was obtained by means of self-report given by the workers and it was measured through the Work Ability Index (WAI)^1^. This index was developed by Finnish researchers and its measurement is based on the workers' self-perception^1^. The WAI was previously translated and adapted to Brazil by researchers from Universities in the state of São Paulo^1^. Afterwards, it was validated by a study realized with workers from an electrical company in the state of São Paulo, Brazil^9^. It is composed by seven aspects: 1) the person's current work ability compared with the best of their life; 2) the work ability compared with the work demands; 3) total number of self-perceived diseases diagnosed by the physician; 4) estimated loss of work due to illness; 5) absence from work due to illness; 6) self-prognosis of work ability; and 7) mental resources. The results can vary between a score of seven to 49 points, in which a score of seven to 27 classified as the group with low work ability, 28 to 36 with moderate work ability, 37 to 43 good, and 44 to 49 with excellent work ability. The results can be used collectively or individually^1^.

Independent variables (e.g., sociodemographic, occupational, health conditions and life habits) included age, color/race, marital status, sex, education, family income, weekly work hours, evening jobs, absenteeism caused by illness, contact with the public, job demands, self-perceived overall health status, dental health status and smoking. These variables were included in data collection instruments and in the following instruments: the Patient Health Questionnaire (PHQ-9)^10^, the International Physical Activity Questionnaire (IPAQ, short)^11^, the Alcohol Use Disorders Identification Test (AUDIT)^12^, the Social Support Survey used in the Medical Outcomes Study (MOS)^13^ and the Swedish Scale of social Demand-Control-Support (DCS)^14^. These instruments were tested and validated for Brazil^12^ ^-^ ^16^.

PHQ-9 is a multipurpose instrument for screening, diagnosing, monitoring and measuring the severity of depression^10^. IPAQ measures health-related physical activity in population. The IPAQ covers four domains of physical activity: work-related, transportation, housework/gardening and leisure-time activity. This questionnaire also includes questions about time spent sitting as an indicator of sedentary behavior^11^. The AUDIT is a very reliable and simple screening tool that is sensitive for early detection of risky and high risk (harmful) drinking. The AUDIT screening procedure in clinical settings is linked to a decision process that includes brief interventions with heavy drinkers, or referral to specialized treatment for patients who show evidence of more serious alcohol involvement^12^. The MOS is a study of variations in physicians' practice styles and patients' outcomes in competing systems of care^13^. Outcomes included clinical endpoints; physical, social, and role functioning in everyday living; patients' perceptions of their general health and well being. The DCS is an instrument for addressing job stress^14^.

All analyses were conducted with the *Statistical Package for the Social Sciences*(r) software and analyzed through descriptive statistics and inferential statistics. The WAI was evaluated through the Mann-Whitney and Kruskal Wallis tests for comparisons between groups. Also were carried out analyses on WAI and quantitative variables, through Spearman's coefficient correlation. Significance level set at 5%. Trained professionals with graduate education guided the interviews. During preliminary meeting for the survey, the research proposal was presented to the participants. Information on the procedures involved in the research activities was also presented to the participants. Participation in the study was formalized through the signing of the separate informed consent form. The Research Ethics Committee of the Federal University of Juiz de Fora approved the study under protocol n. 224/2010.

## Results

### Characterization of study population

 The target study population consisted of 119 workers. 84 workers (70.6%) were excluded because they were away, changed function or were outsourced workers during the period of data collection, getting a sample of 35 (29.4%) workers. There was also a loss of 15 workers who answered incompletely the questionnaire. The remainder 20 workers correspond to 23.8% of 119 workers.

Regarding the personal characteristics of the subjects, all participants were male (100%), 80% were married or in a stable relationship, 75% had the second stage of basic education level. Mean age was 54.9 years (SD=5.7). The age group 51 to 60 years concentrated the largest portion of the population (63.2%). Income range from 5 to less minimum salaries was more prevalent (66.7%) (Table 1).

**Table 1:** Sociodemographic characteristics of the security guards at the Federal University of Juiz de Fora. Juiz de Fora, Minas Gerais, Brazil, 2012-2013

Variables	Category	n	%
Ethnic composition	white	11	55
	black/mixed	9	45
Marital status	married/cohabiting	16	80
	single	4	20
Scholar level	University	5	25
	high school or less	15	75
Household income (minimum wage)	≤ 5	12	66.7
	5 to ≤ 10	5	27.8
	up to 10	1	5.6
Physical activity	sedentary	3	15
	little active	5	25
	active	12	60
Smoking	yes	3	10.5
	no	17	89.5

Differences in totals are explained by the fact that some information regarding a few variables was lost. Data below 10% were not informed.

Related to some characteristics of the lifestyle and health conditions among the participants on social network, when questions about aspects of life with family, friends and group activities were asked, the majority of workers (73.7%) reported having one or more relatives, and 68.4% one or more friends with whom they felt comfortable talking about everything or almost everything. With regard to group activities, most workers (75%) denied participating in sports or artistic activities. In contrast, most claimed to have taken part in other group activities in last year, and 57.9% reported having participated in meetings of neighborhood associations or employees, unions and parties (Table 2).

**Table 2:** Lifestyle and health conditions of the security guards at the Federal University of Juiz de Fora. Juiz de Fora, Minas Gerais, Brazil, 2012-2013

Variables	Category	n	%
Self-assessment of general health	good	16	80
	bad	4	20
Self-assessment of oral health	good	14	73.7
	bad	5	26.3
Depression signs and symptoms	present	1	5.6
	absent	17	94.4
Alcohol dependence	abstinence	12	75
	consumption without risk	3	18.8
	dependence	1	6.3
Relatives with whom feel comfortable talking about everything or almost everything (Social Network)	none	5	26.3
	one or more	14	73.7
Friends with whom feel comfortable talking about everything or almost everything (Social Network)	none	6	31.6
	one or more	13	68.4
Participation in sports activities in group activities or Artistic (Social Network)	no	15	75
	yes	5	25
Sport and physical activity in group (Social Network)	no	8	42.1
	yes	11	57.9
Participation in volunteering and unpaid work (Social Network)	no	8	42.1
	yes	11	57.9
Participation in religious activities (Social Network)	no	3	15
	yes	17	85

Differences in totals are explained by the fact that some information regarding a few variables was lost. Data below 10% were not informed.

In relation to the occupational variables, the average length of service was 31.2 years (SD=5.5) ranging from 22 to 45 years. The majority (95%) had only one job, working 40 hours or less (88.9%) and did not work at night or on shift rotation (75%). The contact with the public was direct for all workers (100%). Regarding demand, control and social support at work, just over half (55%) with high demand and low control (60%); by combining demand and job control, of the four possible combinations the majority (40%) showed passive jobs (low demand/low control). In contrast, 80% of security guards had high social support at work (Table 3).

**Table 3:** Labor characteristics of the security guards at the Federal University of Juiz de Fora, Minas Gerais, Brazil, 2012-2013

Variables	Category	n	%
Service time (years)	minimum	22	-
	maximum	45	-
	media	31.2	-
	dp	5.5	-
Number of jobs	one	19	95
	two or more	1	5
Weekly workload	> 40 horas	2	11.1
	≤ 40 horas	16	88.9
Working at night	yes	5	25
	no	15	75
Social support at work	low	4	20
	high	16	80
Demand-control model	high labour requirement	5	25
	passive job	3	15
	active job	4	20
	low labour requirement	8	40

Differences in totals are explained by the fact that some information regarding a few variables was lost. Data below 10% were not informed.

The average WAI score among workers was 40.7 points (SD=7.2), ranging from 24 to 49 points and the prevalence rates of good work ability was 80% (Table 4).

**Table 4:** Distribution of security guards according to the Work Ability Index (WAI). Juiz de Fora, Minas Gerais, Brazil, 2012-2013

WAI* Classification	Scores	n	%
Low	07-27	2	10
Moderate	28-36	2	10
Good	37-43	8	40
Excellent	44-49	8	40
Total		20	100

*Work Ability Index

It was possible to verify that the highest prevalence rates of good work ability were found among security guards aged between 51 and 60 years old, married and white. Regarding the habits and lifestyles, we found higher prevalence rates of good work ability in individuals who rated their general and oral health as good, in those without depression, in those security guards who were classified as active or very active in physical activity level, practiced abstinence or alcohol consumption without risk, do not smoke, with social support from one or more relatives and friends, and participating in group activities such as meetings, volunteer work and religion activities. With regard to job characteristics, the good work ability was more prevalent among security guards who had only one job, work up to 40 hours per week, did not work at night, had high social support at work and had their functions classified as low requirement job, resulting from the combination with low demand and high control (Table 5).

**Table 5:**
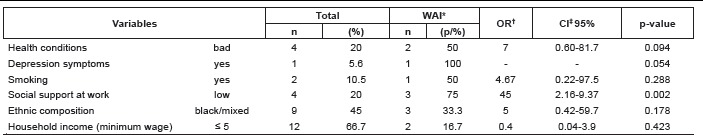
Analysis among sociodemographic and laboral variables on security guards according the Work Ability Index (WAI). Juiz de Fora, Minas Gerais, Brazil, 2012-2013

* Work Ability Index; † Odds Ratio; ‡ Confidence interval.

## Discussion

The aim of this study was to evaluate the work ability and health status on security guards at a public University. In this study the results showed that the studied population presents sociodemographic and occupational characteristics different to the general population, but differed mainly in regard to the type of relationship with the Institution. The security guards had stable job, which is a situation that differs from the growing instability and outsourcing that happens in many sectors in Brazil in recent years, impacting on the working conditions for population, labor relations and generating informal work relations that set up the world of work today. 

But it is important to note that the favorable profile seen in several respects to those workers may be a consequence on healthy worker effect. It is worth noting that the good result for work ability and health profile identified on participating workers may be related to this issue. The healthy worker effect is quoted by various authors that performed studies on work ability and on the profile of health in the workplace, and these authors always stress the importance of carefully analyzing this effect when discussing the results of studies^15^ ^,^ ^17^ ^-^ ^19^.

In the present study, the population was concentrated in the group aged 51 to 60 years. These findings are in line with other authors^20^. They estimated that the segment of workers aged over 50 years is the one that will grow over the coming decades. However, in case of University security guards, what happens is that there is no more public service call for positions for this post; younger security guards are being hired through outsourcing. Thus, the current workers are slowly being replaced by outsourced workers and security guards that are public servants and still in the profession are close to retirement, explaining the particular age profile of this population. While there is consensus that increasing age is associated with decrease in physiological capacity, it is known that the work ability will be affected only if the job performance is dependent on the physiological capacity. In addition, other job characteristics related to the environment or organization can reduce the negative effect of age on work ability^19^. In this study the highest prevalence of good capacity for work was among the individuals aged 51 to 60 years. 

Regarding gender, 100% of security guards were male. This shows that even with the increased participation of women in the labor market, there are functions that are still executed predominantly by men, which can be explained from an historical point of view. It is noteworthy that this feature of our study population is important with regards to work ability, since women have about 2.2 times higher chance to have low work ability in relation to men. Thus, a possible explanation for the fact that 80% of security guards have shown good work ability may be related to gender, because the negative relationship with female work ability is related to the issue of their double workday. Although women have been increasingly entering into the labor market, acquiring autonomy and equal rights with men in several respects, the tasks of caring for the house, children and husband have not ceased being a woman's responsibility and ultimately may have an effect on health status and work ability, a scarce fact in the male population^18^. Regarding ethnic composition, most security guards were white as well as in a other study^6^. However, in both studies this characteristic was not significantly associated with work ability.

Results obtained with PHQ-9 demonstrated that 94.4% of security guards did not show depression symptoms. Thus, the fact that the highest prevalence rates of good work ability were found among these security guards, confirming results showing that individuals with depressive symptoms had 1.2 times greater chance of being incapacitated than those without those symptoms ^14^.

Concerning life habits as smoking, some authors^18^ showed in their study that individuals who did not smoke had higher scores of work ability. In the present study it was observed that the highest prevalence rates of good work ability was found among subjects who did not smoke or had never smoked, but the study done by other authors^21^ demonstrated no significant association between smoking and work ability. When classifying the physical activity level by IPAQ, the higheest prevalence of good work ability (60%) was found among individuals who were active or very active, however there was no significant association between work ability and physical activities^22^ ^-^ ^23^. This finding is supported by other authors^11^ that did not find significant association between physical activity level and the work ability. The authors argued that good physical activity levels do not guarantee the improvement of the work ability.

Regarding network and social support, we found that the highest prevalence rates of good work ability were among the security guards who had social support from relatives and friends and those who reported participating in group activities such as meetings, volunteer work and religious activities. Also, married individuals have higher levels of social support compared to non-married ones. A possible explanation could be that married people have in their partner or spouse a primary source of support. This information may explain the higher prevalence rates of individuals with high social support, since 80% of security guards are married. However, this feature is not often emphasized in the literature when it addresses the work ability^13^.

Another characteristic of workforce extensively studied, both nationally and internationally, may concern the causes of stress and its effects on health. Some authors^24^ argued in their study that decreased job control and increased negative influence of job demands on private life are the most important work factors associated with reduced work ability. Increased job control, and decreased negative influence of job demands on private life are the main factors associated with improved work ability^24^. In this study the highest prevalence rates of good work ability was found among those with low labor requirement, resulting from the combination of low demand and high control. Previous studies showed that this is the ideal type of work, in which a more relaxing and less stressful work environment may allow employees to have higher control over their work^14^. 

Regarding social support at work, this refers to the interaction between colleagues and supervisors in cooperation to get the job done and can contribute to reducing the weathering of the worker and the health risks^14^ ^,^ ^25^. In the present study the highest prevalence rates of good work ability were among the security guards who had high social support at work. Some authors^22^ emphasize that the improvement of work ability is strongly associated with improved relations with the supervisor and organizational process at work. These authors^22^ also consider that social support should be the basis of labor relations and the strategy of social organization in Institutions, because in this way a reduction and even the prevention of work-related stress is achieved. The appreciation of relationships and workplace environment may promote health benefits for workers and their work ability.

Regarding other job characteristics, the highest prevalence rates of good work ability were found among security guards who have only one job, work only 40 hours a week, and do not work in alternating or night shifts. However, by combining these variables with the work ability, we did not find statistically significant differences.

The present study had some limitations related to the cross-sectional design, which are often used to define prevalence and identify associations. However, cross-sectional studies are carried out at one point in time and therefore, this type of study does not give the direction of the sequence of events, turning impossible to infer causality. Another limitation was due to the use of self-report questionnaires, which may lead participants to answer the questions in a more socially desired manner. Besides, answers to questions may also be influenced by several factors such as memory, comprehension ability and interests of participants. Other limitation was the small sample size of the study, which does not allow us to elicit more definitive conclusions. However, few studies discuss the association of work ability among security guards workers in Brazil, and therefore, this study shows the need to deepen the discussion about associated factors with the work ability in a larger population of security guards. Errors are inevitable in almost any epidemiological study, and they may occur even in the best-conducted randomized trial. Thus, our findings should be generalized with caution.

Despite the study limitations, our study emphasizes the importance of assessing the work ability in this population, once 20% of workers had reduced work ability scores. These findings show the need for urgent intervention to prevent permanent illness and early retirement among security guard workers.

## Conclusion

This study showed that many characteristics might affect the work ability scores; however, several of them can be influenced by an indivudual, both with regard to working environment or lifestyle. Considering the results shown above, we note that healthy life habits and a reorganization of work environments should be encouraged. This could lead to improved work ability scores and perhaps prevent early retirements, contributing to reduce social and economic impact in Brazil. In addition, more important than to reduce the social and economic impact, is to improve work ability by encouraging the healthy life habits and a reorganization of work environment, preventing worker illness and improving their quality of work life.

In conclusion, the aspects considered in the present study are elements that can contribute to the conception and development of measures aimed at preserving the work ability, prioritizing the monitoring and control of occupational stress with emphasis on the lifestyle health conditions, consequently, improving the promotion, and the protection of the worker's health.
